# Discordant moderate aortic stenosis: is it clinically important?

**DOI:** 10.1136/openhrt-2021-001749

**Published:** 2021-10-08

**Authors:** Benoy Nalin Shah, Roxy Senior

**Affiliations:** 1 Cardiology, University Hospital Southampton NHS Foundation Trust, Southampton, UK; 2 Department of Cardiology, Royal Brompton Hospital, London, UK

**Keywords:** aortic valve stenosis, echocardiography, heart valve diseases

Quantitative Doppler echocardiography is the gold standard method for assessing the degree of aortic stenosis (AS), which is classified as mild, moderate or severe. This technique was validated in the latter part of the 20th century against the gold standard of that time, cardiac catheterisation derived valve area using the Gorlin hydraulic principle.^1^ In echocardiography, the Doppler parameters used to assess stenosis severity include the peak transvalvular velocity (AV Vmax), mean pressure gradient and the effective orifice area (EOA), determined using the continuity equation.

Assessing AS is straight forward when all parameters indicate the same severity of stenosis—for example, if the AV Vmax, mean gradient and EOA all indicate mild AS—but becomes more challenging when the parameters do not all indicate the same severity—for example, the AV Vmax and mean gradient indicate moderate AS, yet the EOA suggests severe AS. In the former example, the parameters are *concordant* whereas in the latter they are *discordant*.

Over a decade ago, Minners *et al* analysed 3483 echocardiograms to assess the frequency of these inconsistencies and they found that almost a third of patients with severe AS based on EOA (<1.0 cm^2^) had a mean gradient in the moderate AS category (ie, <40 mm Hg).^2^ Furthermore, the authors plotted curves using the original Gorlin formula to assess the relationships between EOA, mean gradient and AV Vmax. They found that an AVA of 1.0 cm^2^ correlated to a mean gradient of 21 mm Hg and an AV Vmax of 3.3 m/s. Conversely, a mean gradient of 40 mm Hg corresponded to an EOA of 0.75 cm^2^ (not 1.0 cm^2^) and an AV Vmax of 4.0 m/s corresponded to an EOA of 0.82 cm^2^. Thus, in their cohort, severe AS was diagnosed in 69% of patients based on EOA but only 45% by AV Vmax and just 40% by mean gradient. They also noted that left ventricular (LV) stroke volume was significantly lower in the patients with discordant parameters.

This followed on from a paper published the previous year by Hachicha *et al*,^3^ in which they found that up to a third of patients with AS and normal LV ejection fraction (EF) had a low flow state, defined as an indexed stroke volume (SVi) <35 mL/m^2^. These observations gave rise to the recognition of the paradoxical scenario in which LV EF is normal (>50%) though transvalvular flow is low—low-flow low-gradient severe AS with normal EF. Assessing flow using the transvalvular flow rate (in which stroke volume is indexed to LV ejection time rather than body surface area) has seen a renewed interest^4 5^ and may help explain some of the inconsistencies in Doppler criteria observed in prior studies.

A retrospective Australian study from Strange *et al* in 2019 shone the spotlight on moderate AS when they found that 1-year and 5-year outcomes were similar in moderate and severe patients with AS.^6^ A recent large echocardiographic analysis reported that only 13% of patients with moderate AS had no other major cardiac abnormalities, such as LV systolic or diastolic dysfunction, mitral/tricuspid valve disease or right ventricular dysfunction.^7^ Thus, moderate AS is rarely found in isolation.

In this issue of *Open Heart*, Pio and colleagues have now examined how frequently discordant Doppler data are found in *moderate* patients with AS.^8^ They performed a retrospective search of patients that underwent echocardiography in two hospitals (in Holland and Singapore) between 2001 and 2018 and identified those with an EOA of 1.0–1.5 cm^2^, which they used as the marker of moderate AS. This revealed 790 patients (mean age 71 years) and the authors found that nearly one in five patients (150/790, 19%) had discordant Doppler parameters—that is, a valve area of 1.0–1.5 cm^2^ but a mean gradient <20 mm Hg. Over a median follow-up period of 4.9 years, the discordant AS cohort were less likely to undergo aortic valve replacement (26.7% vs 44.1%) and had higher mortality (60% vs 43%) compared with patients with concordant moderate AS parameters (ie, AVA 1.0–1.5 cm^2^ and mean gradient >20 mm Hg). On multivariable Cox regression analysis, moderate AS with LV EF <50% was an independent predictor of mortality, irrespective of concordant or discordant AS parameters.

Demographic (patient and clinical) and echocardiographic factors that were independently associated with discordant AS were age, coronary heart disease, LV EF and SVi. Interestingly, although a low flow status (SVi <35 mL/m^2^) was more common in the discordant group, this was only present in 26% cases (vs 1.5% in the concordant group), meaning that roughly three-quarters of the discordant cohort had a normal SVi. Data on transvalvular flow rate are not presented.

What could account for discordant parameters being identified in as many as one in five patients? Several points merit discussion. First, whenever a patient has discordant Doppler parameters of severity, it is crucial to review the echo images looking for potential measurement errors. The most common error is in measurement of the left ventricular outflow tract (LVOT) diameter, which is used to derive an LVOT area for the continuity equation and it is known that transthoracic echocardiography (TTE) frequently under-estimates this area compared with cross-sectional imaging modalities. Importantly, the way in which the LVOT diameter should be measured changed during the lengthy period of this study. Previously, it was recommended to measure the LVOT diameter approximately 0.5–1.0 cm below the valve leaflets in the parasternal long axis view. However, as it became apparent that this frequently under-estimated the true LVOT area, practice was changed and now measurement is recommended at the hinge points (base) of the leaflets. Thus, many of the echocardiograms included in this study *may* have had an erroneously low aortic valve area due to a small LVOT area and, in fact, the mean gradient <20 mm Hg was a correct marker of mild AS only.

**Figure 1:**
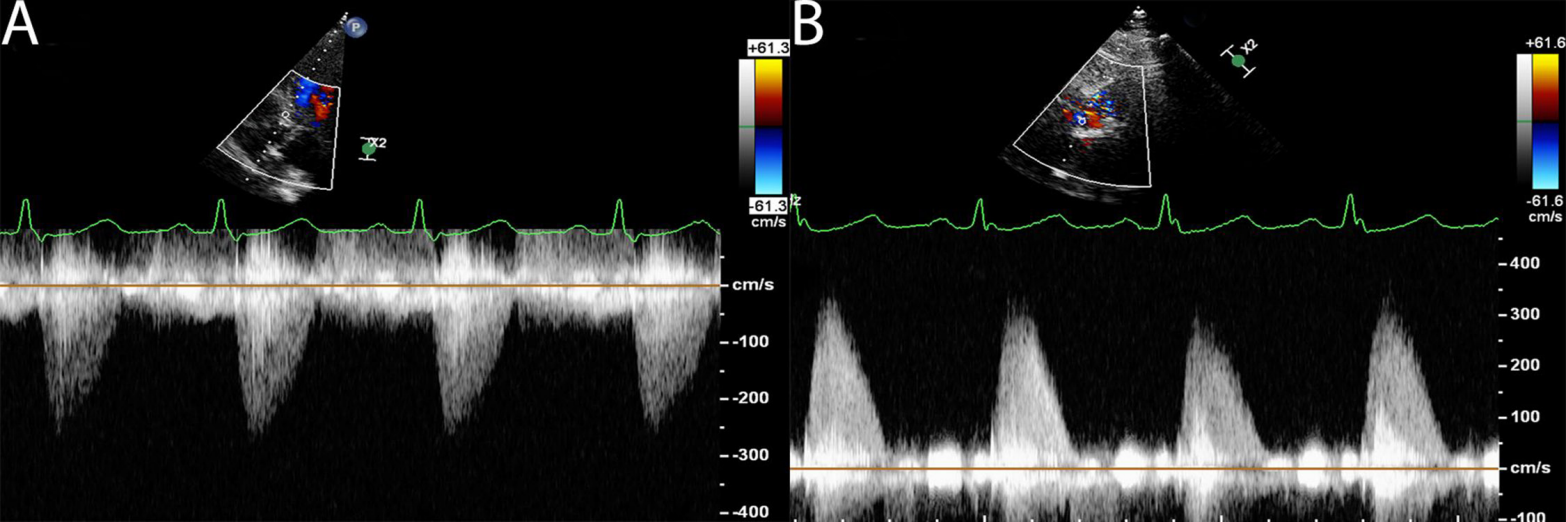
Apical 5-chamber (AP5Ch—(A)) and right sternal edge (RSE—(B)) continuous wave spectral Doppler tracings from a patient with aortic stenosis. Left ventricular outflow tract (LVOT) diameter was 2.1 cm and LVOT velocity time integral (VTI) 22 cm. Peak transvalvular velocity, mean gradient and effective orifice area were 2.6 m/s, 13 mm Hg and 1.40 cm^2^, respectively from the AP5Ch view but 3.4 m/s, 21 mm Hg and 1.05 cm^2^, respectively using RSE Doppler data. Thus, *discordant* moderate aortic stenosis (AS) became *concordant* moderate AS using the non-apical continuous wave Doppler data.

Second, when assessing AS, multiple insonation windows should be used to obtain the maximum transvalvular velocity. This frequently necessitates the suprasternal and right parasternal windows—and sometimes even the right supraclavicular fossa or subcostal views—but none of these were apparently used in the current study (the Methods section states that apical 5-chamber and 3-chamber views only were used for Doppler assessment). It also appears that the PEDOF probe was not used in these studies either, which can also increase the peak velocities recorded. These alternate windows and probe techniques are not only applicable to patients with suspected severe AS—as the image in figure 1 shows, these techniques can also be of great help with lesser degrees of stenosis severity and should be used as a routine in *all* patients with aortic stenosis (time pressures frequently prevent this).

Third, it is not clear how patients in atrial fibrillation were assessed. Atrial fibrillation was prevalent in these patients (213/790, 27%) and practice can vary from taking an average of 3 beats to an average of 5 beats to using a peak pulsed wave Doppler and a peak continuous wave Doppler tracing. Variation in practice for AF patients between the two centres (in different continents over a length period of time) is likely and may account for some of these differences.

However, in many respects, these findings also should not come as a significant surprise. Prior studies have highlighted the shortcomings of our current severity grading system. Considerable research into the factors associated with discrepant AS parameters has made the assessment of AS severity increasingly challenging and has led to a renaissance of interest in parameters such as the transvalvular flow rate (FR). In a post-hoc analysis of the Simvastatin and Ezetimibe in Aortic Stenosis trial, where patients with mild-moderate patients with AS were enrolled, discordant AS was observed in 28% of the population and almost 60% of these patients had low transvalvular FR.^4^ In patients with low transvalvular flow rate, 28% demonstrated normal SVi. Unfortunately, the current study does not include flow rate data, but one would hypothesise that the discordant group were more likely to have a significantly higher prevalence of low flow rate compared with low SVi.

The prognostic data are of great interest; we note that discordant moderate AS per se did not affect survival but did so in association with low LV EF. Although a much-maligned parameter in recent years, these data underscore the oft-seen prognostic power of reduced LV EF. Furthermore, SVi was not associated with mortality—in the aforementioned study transvalvular flow rate, but not SVi, predicted mortality in moderate patients with AS.^4^

In summary, these data highlight that discrepancies between Doppler indices of stenosis severity are not restricted to patients with severe AS (as judged by EOA), but lesser degrees of stenosis severity also. Care should be taken to ensure high-quality studies are performed and to eliminate errors in measurements before diagnosing discordant AS. Low transvalvular flow rate and LV systolic dysfunction are markers of poor outcome.
